# The Effect of Diazinon on Cell Proliferation and Apoptosis in Testicular Tissue of Rats and The Protective Effect of Vitamin E

**DOI:** 10.22074/ijfs.2019.5612

**Published:** 2019-04-27

**Authors:** Fatemeh Rahimi Anbarkeh, Mohammad Reza Nikravesh, Mehdi Jalali, Hamid Reza Sadeghnia, Zinat Sargazi

**Affiliations:** 1 Department of Anatomy and Cell Biology, Faculty of Medicine, Mashhad University of Medical Sciences, Mashhad, Iran; 2 Department of Pharmacology, Faculty of Medicine, Mashhad University of Medical Sciences, Mashhad, Iran; 3 Research Center for Neuroscience, Torbat Heydariyeh University of Medical Sciences, Torbat Heydariyeh, Iran

**Keywords:** Apoptosis, Diazinon, Proliferation, Testis, Vitamin E

## Abstract

**Background:**

Diazinon (DZN) is an organophosphate pesticide, and nowadays this pesticide is mostly used in agri- culture. In this study, we analyzed the effects of DZN and vitamin E (Vit E) on apoptosis and the proliferation of germ cells in rat testis.

**Materials and Methods:**

In this experimental study, 30 male Wistar rats were divided into five groups (n=6 per group) consisting of control, sham (received olive oil), experimental group i (60 mg/kg DZN), experimental group ii (60 mg/kg DZN and 200 mg/kg Vit E), and experimental group iii (200 mg/kg Vit E). After six weeks, left testis of rats was removed for the detection of proliferative cell nuclear antigen (PCNA) and terminal deoxynucleotidyl transferase end-labeling (TUNEL).

**Results:**

Compared with the control group, DZN in the experimental group i decreased the number of PCNA-positive cells and increased the number of TUNEL-positive cells (P<0.001). Vit E improved detrimental changes by the de- crease in the rate of apoptosis and the increase in the proliferation of testicular germ cells (P<0.001).

**Conclusion:**

Vit E can decrease the number of TUNEL-positive cells and increase the number of PCNA-positive cells by the neutralization of the toxicity caused by DZN in the testicular tissue.

## Introduction

Pesticides are widely used in agricultural production to prevent or control pests, diseases, weeds, and other plant pathogens in an effort to reduce or eliminate yield losses and maintain high product quality. Despite their popularity and extensive use, pesticides have raised serious concerns about human health arising from the exposure of farmers when mixing and applying pesticides or working in treated fields and from residues on food and in drinking water for the general population. The wide usage of pesticides in agriculture and general hygiene causes serious problems in ecosystem and hygiene risks including acute, sub-acute, and chronic human and animal poisoning and therefore it is an important concern (1, 2).

Because of the non-polar and lipophilic structure of organophosphates, they are absorbed quickly after eating or breathing, and after the absorption, they aggregate in adipose tissues, kidney, liver, and salivary glands. The long-term exposure of diazinon (DZN) to the skin can cause severe poisoning. Organophosphates undergo different metabolic reactions, and finally, their metabolites are excreted by urine, faces, and expiration. Some of the poisonous metabolites are accumulated in adipose tissue requiring long time periods to be excreted from the body (3).

DZN is one of the most well-known organophosphate pesticides which is synthetic and not naturally found in the environment. It is more common to be used for paddies, fruit trees, and ectoparasites of the livestock. DZN activity is similar to other organophosphates which are able to block acetylcholinesterase enzyme. These compounds can bind to some enzymes in the human body; however, its effects on acetylcholinesterase blockade has been clinically important (4). DZN causes pathological changes in the human body such as hematological and reproductive disorders, as well as kidney, liver, cardiovascular, and the central nervous system (CNS) toxicity through an elevation in oxidative stress and free radicals (5, 6). The toxicity of organophosphate compounds are not confined to inhibit acetylcholine enzyme; rather, they are capable of inducing programmed cell death via internal and external apoptosis pathway (7). Moreover, the induction of apoptosis in various tissues of the body is mediated by the activation of caspase-dependent pathways leading to cell death (8). Spermatogenesis is a process in which immature germ cells are matured to mature cells in testicular tubes (9). According to Dadhich et al. (10) apoptosis and cell proliferation play important roles in controlling the number of testicular cells thereby a hormonally controlled process that precisely regulates the balance between the generation of Sertoli and germ cells. Apoptosis has two main roles in the normal spermatogenesis namely, decreasing the number of germ cells that can be supported by Sertoli cells and the removal of abnormal sperms by which ineffective cells such as old, immature, and damaged cells are omitted (10, 11).

DZN can alter the diameter of seminiferous tubules by loosening the connective tissue and the muscles around them. Moreover, the size of the germinal cells in response to intoxication with DZN changes and becomes smaller than their normal counterparts (12). Vitamin E (Vit E) as a lipid-soluble antioxidant inhibits lipid peroxidation thereby maintaining the integrity of the cell membrane. Regarding a study conducted by John et al. (13) Vit E ameliorated (not blocked) organophosphate-induced oxidative stress by decreasing lipid peroxidation and altering antioxidant defense system in erythrocytes. Although the antioxidant and anti-apoptotic effects of Vit E have been shown in a vast number of studies, no study has been so far performed to study the effect of this vitamin on testicle toxicity caused by DZN. Hence, in this study, the apoptosis and proliferation rate of germ cells in testicles of male rats intoxicated with DZN were examined, and the protective impacts of Vit E administration were assessed.

## Materials and Methods

### Animals

In this experimental study, thirty adult male Wistar rats weighing 200-250 g were procured from the Animal House of the Medical University of Mashhad. They were housed in the standard situation (six animals per cage) at a temperature of 22 ± 2°C and 12:12 hour light dark cycle. The animals had free access to food and tap water during the experiment. Rats were randomly divided into five groups as follows: i. Control group that received no therapy or drug solvent, ii. Experimental group i that received 60 mg/kg DZN, dissolved in olive oil and intraperitoneally administered, iii. Experimental group ii that received 60 mg/kg DZN along with 200 mg/kg Vit E by intraperitoneal injection and daily gavage, respectively, iv. Experimental group iii that received 200 mg/kg Vit E by daily gavage, and v. Sham group that received pure olive oil as an intraperitoneal injection (IP).

After six weeks, rats were anesthetized, and their testicles were removed for further study. This study was carried out in the Histology Laboratory of the Medical University of Mashhad and approved by the Ethics Committee of Mashhad University of Medical Sciences (no.911096).

### Chemicals

DZN 98% was purchased from Ariashimi Company. TUNEL assay kit was procured from Roche, Germany. PCNA kit was purchased from Zymed Company. Vit E (α-tocopherol acetate) was procured from Sigma.

### Testicular perfusion protocol

**Fig 1:**
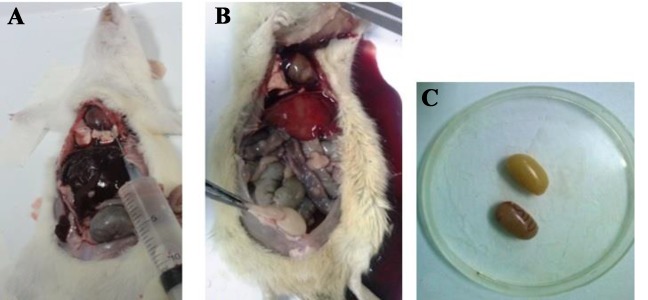
Perfusion phase through the left ventricle. **A.** The Pre-perfusion stage while the gut had a normal appearance, **B.** Post-perfusion characterized by a change in the appearance of internal organs such as intestine, liver, and testicles, showing that perfusion was successful and then the initial fixation happened, and **C.** The testis color changed after the perfusion process.

Tissue perfusion is necessary for the drainage the blood from tissues, and it enhances the background and the quality of staining. To do perfusion, first, the animals were anesthetized by ether and their thorax was opened through the surgical procedure. Perfusion was carried out by the injection of rinse and fixative solutions (by 10 or 20 ml syringes) into the left ventricle. During the opening of the thorax, an incision was made in the midline to minimize the damage to the vascular walls. Therefore, the bleeding and rupture of the vessels were reduced. The joints of the heart along with their coatings were separated from the bone. The right atrium was perforated with a needle while another syringe needle was inserted into the left ventricle and rinse solution was injected until drained out from the right atrium hole and become clear. Then, the fixative solution was injected promptly, and this procedure continued until the hindlimbs and forelimbs of the animals became pale and started to shake. After these signs, the left testicle was removed to perform further procedures as shown in Figure 1 (14).

### Sample preparation

To prevent autolysis by lysosomal enzymes and tissue damage by bacteria, as well as to maintain the structural features and the ingredient volume of the tissue, testicles were fixed by 4% paraformaldehyde for 72 hours (considering the size, type of samples, and the fixative quality). After the fixation process, to prepare the tissue for the examination under an optical microscope, the tissue processing was carried out. Finally, the samples were paraffin-embedded. Then, the samples were cut by a microtome apparatus at 5 μm, and the slides were placed on poly-L-lysine slides. After deparaffinization and hydration by alcohol with descending gradient, samples were examined by PCNA and TUNEL techniques under an optical microscope.

### TUNEL assay

The apoptosis rate was evaluated by the TUNEL assay as the staining of apoptotic cells was performed based upon the following protocol (15): samples were deparaffinized with xylol in two steps (5 minutes for each step), placed in ethanol with decreasing gradient as 95, 80, and 70% and washed three times with phosphate-buffered salin (PBS). After washing the slides with PBS three times, the slides were incubated with 50 μL protein kinase K and rinsed three times by PBS. Then, the slides were incubated with TUNEL reaction mixture for 24 hours at 4°C. Samples were then incubated with anti-fluorescein antibody conjugated with horse-radish peroxidase (Converter-POD) and stained with chromogenic diaminobenzidine (DAB).

Control slides: a few numbers of slides were incubated with DNase I solution and rinsed with PBS. The slides were stained according to the previous steps of staining. Since DNase I enzyme causes DNA fragmentation, the stained slides were considered the positive controls. The negative control slides were stained with the omission of terminal transferase.

### Cell proliferation examination method

The immunostaining was performed using the PCNA kit and based on the protocol, as previously described (16): this technique is similar to TUNEL. The peroxidase activity within the tissue was blocked by the addition of 3% hydrogen peroxide in methyl alcohol. Then, a few drops of blocking solution were poured (100 μl) on the samples. Next, the samples were incubated with the anti-PCNA biotin-conjugated antibody for 30 to 60 minutes in the humid environment and at room temperature. Finally, the samples were stained with chromogenic DAB. To perform hematoxylin staining, samples were incubated with Alcian blue (or hematoxylin), rinsed with running buffer, and then washed with distilled water. Then, the samples were dehydrated with an increasing gradient of ethanol. Xylene was applied for the transparency of the samples in two steps, and then they were mounted using special glue.

Control slides: some positive control slides (according to the materials of the kit) were stained. The negative control slides were also stained after the omission of antibody.

### Stereology method

The numerical density of PCNA-positive and TUNEL-positive cells is calculated in the unit of the surface by unbiased frames grades. For this aim, the testicular sections from different groups were imaged by Olympus optical microscope model BX51. Then, the numerical density of TUNEL- and PCNA-positive cells was calculated in the unit of surface. The average cells in the unit of surface of the rats’ testicles in different groups are calculated based on this formula (15):

Na=ΣQa/fΣP

In this formula, ΣQ is the number of calculated cells, a/f is the area of each frame, and ΣP is the number of clash points in the frame area.

### Statistical analysis

Data were analyzed using the SPSS 16 software. Results were expressed as the means and standard deviations (means ± SD). Statistical analysis was performed with one-way ANOVA followed by Tukey’s test to compare the differences between groups. Differences were considered statistically significant if P<0.05.

## Results

### Effect of Vitamin E on cell apoptosis in testis tissue following exposure to diazinon

The effects of DZN and Vit E on cell apoptosis are shown in Table 1. The histological study using the TUNEL assay showed that DZN in the experimental group i significantly increased (P<0.001) spermatogonial cells and the rate of apoptosis in primary spermatocytes compared to the control group (Figes.2, 3). The rate of apoptosis was decreased in the experimental group ii, which shows the protective role of Vit E. In the sham and experimental group iii, the number of apoptotic cells was similar to the control group and most of their seminiferous tubules lacked apoptotic cells, and only one or two apoptotic cells was observed in some tubules (P=1.000, Fig .3).

**Table 1:** The effect of DZN and Vit E on PCNA- and TUNEL-positive cells in testes tissue of rats


Group	PCNA positive cells		TUNEL positive cells	
		Spermatogonia	Primary spermatocyte	Spermatogonia	Primary spermatocyte

Control	0.32320 ± 0.027	0.35834 ± 0.037	0.0450 ± 0.011	0.0896 ± 0.014
DZN	0.04989 ± 0.011^a^	0.05888 ± 0.015^a^	0.2174 ± 0.024^a^	0.4241 ± 0.052^a^
DZN+Vit E	0.15868 ± 0.016^a, b^	0.20384 ± 0.026^a, b^	0.1092 ± 0.025^a, b^	0.2008 ± 0.014^a, b^
Vit E	0.33266 ± 0.032	0.36034 ± 0.037	0.0517 ± 0.010	0.0958 ± 0.011
Sham	0.31743 ± 0.031	0.35734 ± 0.037	0.0375 ± 0.010	0.0827 ± 0.012


Values are expressed mean ± SD. ^a^; Significantly different from control group (P<0.001), ^b^; Significantly different from DZN-group (P<0.001), DZN; Diazinon, Vit E; Vitamin E, PCNA; Proliferative cell nuclear antigen, and TUNEL; Terminal deoxynucleotidyl transferase end-labeling.

**Fig 2:**
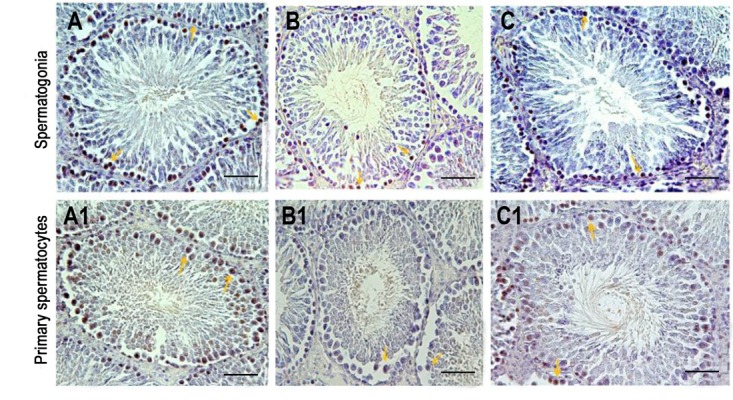
Testicular tissue sections after the TUNEL assay for the detection of apoptosis in primary spermatocytes and spermatogonial cells. **A, A1.** Control group. Most of the seminiferous tubules did not undergo apoptosis, **B, B1.** In the experimental group i. DZN caused the increase in the apoptotic cells compared to the control group, and **C, C1.** In the experimental group ii, vitamin E decreased the number of apoptotic cells compared to the experimental group i. The TUNEL-positive cells are marked by an arrow (scale bar: A, B, C: 5 μm, A1, B1, C1: 10 μm). TUNEL; TUNEL; Terminal deoxynucleotidyl transferase end-labeling and DZN; Diazinon.

**Fig 3:**
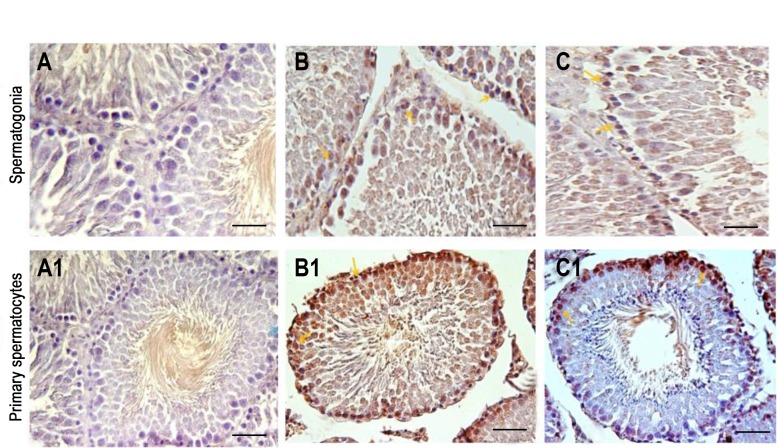
The effect of DZN and Vit E on the apoptosis rate of primary spermatocytes and spermatogonial cell in seminiferous tubules. The results were presented as the mean ± SD. DZN; Diazinon, Vit E; Vitamin E, ***; Compared to the control group (P<0.001), ###; Compared to the DZN-group (P<0.001).

**Fig 4:**
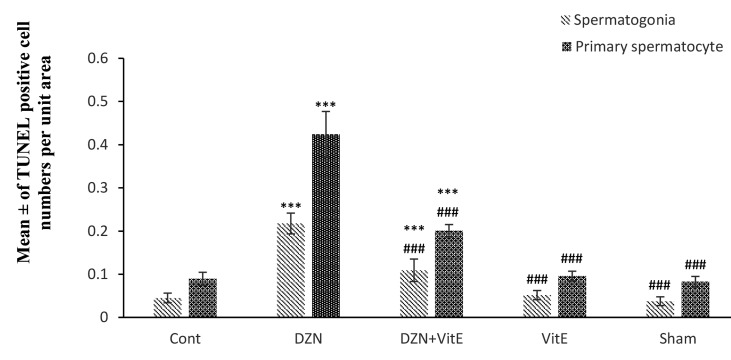
Testicular tissue sections after the PCNA technique. **A, A1.** The control group. Most of the spermatogonial cells and primary spermatocytes are proliferating cells, **B, B1.** In the experimental group i, DZN caused a decrease in the proliferation rate of the cells, and **C, C1.** In the experimental group ii, which was receiving DZN and vitamin E, the number of proliferating spermatogonia and primary spermatocytes were increased compared to the experimental group i. PCNA-positive cells are determined by the arrow (scale bar:10 μm). PCNA; Proliferative cell nuclear antigen and DZN; Diazinon.

### Effect of Vitamin E on cell proliferation in testis tissue following exposure to diazinon

**Fig 5:**
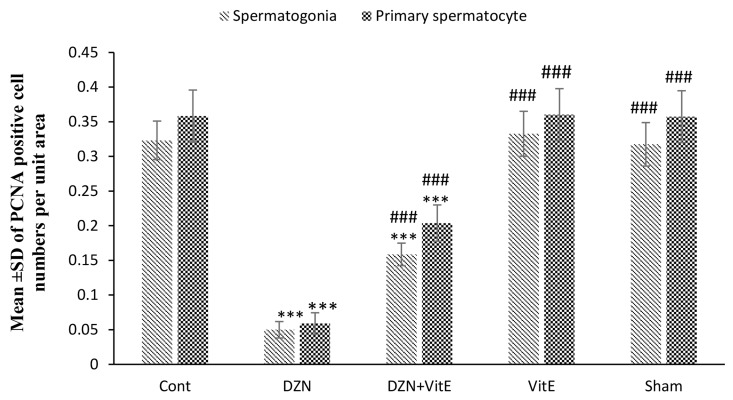
The effect of DZN and Vit E on the proliferation of primary spermatocytes and spermatogonial cells in seminiferous tubules (mean ± SD). DZN; Diazinon, Vit E; Vitamin E, ***; Compared to the control group (P<0.001), and ###; Compared to the DZN-treated group (P<0.001).

The results of DZN on cell proliferation and the protective effect of Vit E are shown in Table 1. Figure 4 indicated the cell proliferation by the PCNA technique. The exposure to DZN in the experimental group i significantly decreased spermatogonial cells and the proliferation of primary spermatocytes compared to control group (P<0.001) and therefore this group had the lowest number of cell proliferation (Fig .5). Vit E, in the experimental group ii, caused the higher rate of proliferation in spermatogonial cells and the primary spermatocytes compared to the experimental group i (P<0.001). As shown in Figure 5, the number of proliferating cells in the control, sham, and experimental group iii were not significantly different (P=0.992).

## Discussion

Several studies conducted on various organophosphates have shown that oxidative stress can cause apoptosis. In this study, the TUNEL assay was employed for the investigation of the effect of DZN on apoptosis of spermatogonial cells and primitive spermatocytes. Histological studies also showed that DZN increased the apoptosis rate of spermatogonial cells and the primary spermatocytes. The administration of Vit E in DZN-treated rats reduced the number of apoptotic cells. Although a majority of absorbed toxins are detoxified and excreted via the detoxification process performed by the liver; however, a portion of toxic compounds may remain and accumulate in different parts of body tissues such as ovary and testis, and therefore affect the reproduction of the animals (17). According to the study performed by Sargazi et al. (15) DZN induced apoptosis in secondary and Graafian follicles while Vit E inhibited DZN-induced apoptosis. In a study carried out by Bustos-Obregon and Gonzalez-Hormazábal (18), they showed that malathion causes apoptosis in type A and B spermatogonia, spermatocyte, and spermatid. Malathion also significantly decreases the activity of serum acetylcholinesterase. According to the study of Razavi et al. (19) sub-chronic exposure to DZN induced caspase-mediated apoptosis, and crocin reduced the toxic effects of DZN by inhibiting apoptosis in aortic tissue. It is also reported that methyl parathion, dichlorvos, and chlorpyrifos increase caspases-3 and -9 in some tissues such as endometrium and retina, and the consumption of vitamin C and E together reduces the pathological effects of DZN and the rate of apoptosis caused by these pesticides (20, 21). Apoptosis is an energy-dependent process that causes the activation of caspases. The transfer of phosphatidyl serine to the outside of the plasma membrane, the changes in the permeability of the mitochondrial membrane, activation and the transfer of caspases to the nucleus, and fragmentation of DNA are the characteristics of apoptosis (22). Also, based on several studies, organophosphates change the gene expression level of gene-related apoptosis including pro-apoptotic Bax and anti-apoptotic Bcl-2 (23, 24). Therefore, organophosphates stimulate apoptosis through the activation of internal and external pathways (7, 8).

As mentioned previously, cellular damages caused by organophosphate compounds have different features, but these detrimental changes mainly affect the structure and the performance of DNA (25). Pina-Guzman et al. (26) confirmed that the toxicity of organophosphate is mediated by phosphorylation of some proteins such as nuclear proteins. Organophosphates have alkylation characteristics which can affect DNA. They also have electrophilic characteristics which can affect nuclear proteins. Therefore, organophosphates including DZN make changes in the chromatin structure and sperm DNA in the spermatogenesis process thereby phosphorylation of nuclear proteins (protamine). According to the research performed by Uzun et al. (27), sperm counts, sperm motility, plasma follicle stimulating hormon (FSH), luteinizing hormon (LH) and testosterone levels were decreased and abnormal sperm numbers were increased in rat testicles. Vitamins E and C had a protective effect on sperm counts, sperm motility and abnormal sperm numbers, but not on plasma FSH, LH and testosterone levels. Correspondingly, organophosphates can affect the normal characteristics of germ cells and their nucleus (28). Therefore, the free radicals produced during the DZN metabolism in the liver can reduce the efficiency of the testicles in the synthesis of hormones and produce low quality germ cells by the elevation of lipid peroxidation and the damage to the cell membrane (12). Vit E can prevent the peroxidation process in biological systems by blocking free radicals. The differences in the type and amount of compounds, as well as the time periods, are different factors that have been considered.

One of the functions of proliferator cell nuclear antigen is the process of DNA polymerase delta and epsilon. PCNA also interacts with other proteins, and by interaction with cyclin-dependent kinases affects the cell cycle. Testicular tissues possess spermatogenetic epithelium in which the germ cells are transferred from margin to the lumen side and developed from spermatogonia to sperm. PCNA expression has a direct association with mitosis. Since spermatogonial cells and spermatocytes are actively dividing in the seminiferous tubules, it could be considered a cellular proliferation marker for the identification of mitotic cells in seminiferous tubules (29-32). The PCNA method showed a significant reduction in cell proliferation in the experimental group i and the increase in spermatogonial proliferation activity and the primary spermatocyte in the control group, sham and experimental group iii. Also, the administration of Vit E in DZN-treated rats significantly increased the number of spermatogonia and spermatocyte proliferation. Organophosphate compounds along with their metabolites exert their toxicity at micromolar concentrations when used in vitro thereby blocking the synthesis of DNA in glial and neuronal cells (33). DZN-induced decreased cell proliferation showed that organophosphates affect cellular responses to cytokine and the ability of cellular proliferation and gene expression by the elevation in the generation of free radicals. Increasing malondialdehyde level may also cause cellular changes and apoptosis (34). Čolović et al. (35) have shown that DZN at different doses decreased the proliferation of blood lymphocytes and the fibroblasts of human skin in the culture medium and also blocked the acetylcholinesterase activity and increased the malondialdehyde level. Penna-Videau et al. (36) reported that one dose of malathion increased the apoptosis rate of germ cells in seminiferous tubules and decreased the number of sperms and activity of plasma cholinesterase. Therefore, DZN and other organophosphate compounds can damage to testicular tissue by the influence on the different cellular processes such as DNA transcription, breaking DNA or proteins chemical bond, decreasing the cells proliferation, and eventually causing the mutation in spermatozoa by the alteration of the gene contents in spermatocyte (37). Vit E has antioxidant properties and can restrict the activity of free radicals and the rate of lipid peroxidation in the cell membrane; thus reducing the damage to testes and spermatozoa (13). According to the results of the current study and previous reports, the use of other antioxidants and the examination of other specific parts of testicular tissue including extracellular matrix proteins are recommended to identify other mechanisms of toxicity.

## Conclusion

DZN caused testicular toxicity by reducing proliferation and increasing apoptosis in testicular germ cells. Vit E could be considered a potential therapeutic agent for testicular toxicity caused by reduced cell proliferation and increased apoptosis. Therefore, Vit E can protect testicular tissues against DZN toxicity. Considering that organophosphates are inevitably used for the quality of agricultural products the use of antioxidant compounds is recommended for those in contact with these toxic agents.
